# Forensic mental health professionals’ perceptions of their dual loyalty conflict: findings from a qualitative study

**DOI:** 10.1186/s12910-021-00688-2

**Published:** 2021-09-16

**Authors:** Helene Merkt, Sophie Haesen, Ariel Eytan, Elmar Habermeyer, Marcelo F. Aebi, Bernice Elger, Tenzin Wangmo

**Affiliations:** 1 Institute for Biomedical Ethics, University Basel, Basel, Switzerland; 2 Service des mesures institutionnelles, Hôpitaux Universitaires de Genève, Geneva, Switzerland; 3 Psychiatrische Universitätsklinik Zürich, Klinik für Forensische Psychiatrie, Zürich, Switzerland; 4 School of Criminal Sciences, University of Lausanne, Lausanne, Switzerland

**Keywords:** Dual role, Dual loyalty, Triangular relationship, Prison, Offender, Qualitative, Therapeutic alliance, Therapeutic relationship, Transparency, Limited confidentiality, Coercion

## Abstract

**Background:**

Mental health professionals (MHP) working in court-mandated treatment settings face ethical dilemmas due to their dual role in assuring their patient’s well-being while guaranteeing the security of the population. Clear practical guidelines to support these MHPs’ decision-making are lacking, amongst others, due to the ethical conflicts within this field. This qualitative interview study contributes to the much-needed empirical research on how MHPs resolve these ethical conflicts in daily clinical practice.

**Methods:**

31 MHPs working in court-mandated treatment settings were interviewed. The interviews were semi-structured and our in-depth analysis followed the thematic analysis approach.

**Results:**

We first outline how mental health professionals perceive their dual loyalty conflict and how they describe their affiliations with the medical and the justice system. Our findings indicate that this positioning was influenced by situational factors, drawing the MHPs at times closer to the caring or controlling poles. Second, our results illustrate how participating MHPs solve their dual loyalty conflict. Participants considered central to motivate the patient, to see the benefits of treatment and its goals. Further, transparent communication with patients and representatives of the justice system was highlighted as key to develop a trustful relationship with the patient and to manage the influences from the different players involved.

**Conclusions:**

Even though individual positioning and opinions towards dealing with the influences of the justice system varied, the results of our research show that, in spite of varying positions, the underlying practice is not very different across participating MHPs. Several techniques that allow developing a high-quality therapeutic alliance with the patient are key elements of general psychotherapy. Transparency appears as the crucial factor when communicating with the patient and with representatives of the justice system. More specifically, patients need to be informed since the beginning of therapy about the limits of medical confidentiality. It is also recommended to develop guidelines that define the level of detailed information that should be disclosed when communicating with the authorities of the justice system.

**Supplementary Information:**

The online version contains supplementary material available at 10.1186/s12910-021-00688-2.

## Background

Mental health care professionals (MHPs) working in court-mandated treatment settings face ethical dilemmas because they are placed in a triangular relationship that involves them, their patients, and the judicial system [1, 2]. The work of the MHPs is not only to care for the patient’s mental health but also to assure public security. Hence, they play a double role, which forces them to find a balance between the individual patient’s rights and ensuring the safety of the general population [3]. Consequently, they face moral dilemmas towards ensuring the rights of their patients within a healthcare setting as well as caring for the welfare of the public who may be harmed should their patient be released into the community—which we refer to as *dual loyalty conflicts—*in their daily practice [1].

Different dual loyalty conflicts arise depending on the mental health professional’s task. The first distinction can be made between forensic mental health professionals acting as experts at court proceedings and clinicians in therapeutic roles. It is argued that these professional roles result in differing ethical conflicts and therefore require separate ethical guidelines [4]. For instance, in the therapeutic context, principles such as beneficence and non-maleficence apply to the patient-physician relationship, in prison as much as in the community [5]. Appelbaum [6] argues that these concepts can not be applied to forensic assessments in court proceedings. In contrast, he considers the principles of truth telling and respect for persons should be the ethical underpinnings. As different set of norms apply to different roles, it is recommended that the roles should be strictly separated to different professionals [7]. Further, even though similar principles apply to the physician–patient relationship in the therapeutic context, mental health professionals working in the criminal justice system face certain role conflicts. Bonner and Vandecreek [8] named typical scenarios such as being asked to alter clinical evaluations, to release confidential information, or to house an incarcerated person in segregation for “psych review”. Further, court-mandated treatment settings present a specific challenge to mental health professionals because their role to treat and to evaluate subside. This paper focuses on the ethical dilemmas that arise in court-mandated treatment settings for persons deprived of liberty, which will be discussed in more detail in the following paragraphs.

Dual loyalty conflicts have the potential to compromise a mental health professional’s behavior but also to infringe on the human rights of the patients undergoing treatment [9]. In fact, mandated treatment settings have repeatedly been criticized for violations of patient’s rights [3, 10]. A violation that is often mentioned refers to medical confidentiality, which is broken when health professionals share information with representatives of the justice system. In particular, it must be possible for the supervisory authority to review the therapeutic procedure or the progress achieved. For this control function, which is ultimately also exercised in the patient's interest, the reports made for the review are of great importance, but these reports could contain confidential information.

The way in which confidentiality is handled by mental health professionals in the correctional context varies from a complete break of it to almost no information sharing [11]. This contrast is found not only in daily practice but also in international guidelines. It is true that the majority of international guidelines recommend that healthcare should be provided in complete loyalty to the mandated patients (see [12]); however, several guidelines such as those of Physicians for Human Rights [13] and the Penal Reform International [14] state that, if the role conflict is previously explained to the patient, then it is morally and legally acceptable to break confidentiality. In the same line, a qualitative study conducted in Switzerland found that mental health professionals regularly inform their patients that confidentiality will be breached when care is provided within the context of a court-ordered therapy [15, 16].

In practice, the field of forensic psychiatry is subject to sets of norms that emanate from two distinct state institutions—the health and judicial system. These two sets of norms use different nomenclatures and therefore they are incommensurable. This is particularly true, as pointed out by Lau and Sachs [17], when one tries to apply the principle of medical confidentiality in court-mandated settings. Confronted with this problem, therapists would take advantage of a harmonized set of guidelines to be applied in court-mandated settings.

In fact, what is under debate is which guiding principles should be applied within this context. In the same spirit, experts have pointed out the lack of shared underlying normative ethical guidelines [1, 18], formalized training, and institutional mechanisms to guide mental health professionals in dealing with potential conflicts of interests [9]. In particular, Niveau and Welle [1] consider that the field of forensic psychiatry has two conflicting ethics, meaning that the MHP’s behavior is influenced by moral principles originating from the two distinct state institutions, and no clear directives on how to solve their incompatibilities (please see for example [1, 6] for a detailed discussion of the ethical clash). In sum, it is widely known that mental health professionals working in mandated treatment settings regularly face ethical dilemmas, and that there is no clear guidance to support their decision-making.

Despite these controversies, mandated treatments are still common practice in at least 75 jurisdictions across the world [3]. In Switzerland, as the criminal code has a system of measures for dangerous and mentally ill persons, the number of ill individuals sentenced to mandated treatments is necessarily higher than in countries that consider them as criminally irresponsible and treat them outside the criminal justice system. At the same time, it is a particularly interesting location for forensic psychiatry, as the perceived set of norms linked to this profession are supposed to be drastically different between the French- and German-speaking language regions [19]. In terms of a potential violation of the patient’s rights by disclosing information about the treatment, practitioners from the French speaking region feel that they are bound to the patient only, while clinicians from the German-speaking region are more closely affiliated with the justice system. Thus, a crack is running through forensic and correctional practice [20] which is thought to show itself clearly at the language barrier. However, this difference could be due to a different ideology (*Weltanschauung)*, which can be traced back to the differences in the catholic and the protestant ethics, famously identified by Weber [21]. The protestant work ethic can be perceived in the alignment of most German speaking practitioners with the criminal justice system, while the French speaking ones choose the side of the patient, in what can be seen as an analogy of the position of both ethics toward the poor and the State. In that perspective, the “Catholic principle of tolerating a lesser evil for the sake of a greater good” [22] seems to justify hiding information to the criminal justice system in order to protect the patient.

Regardless of the causes of these different approaches, mental health professionals face dual loyalty issues in their daily practice. Little is known about the strategies they use to deal with these conflicts. The aim of this qualitative interview study is to start filling that gap by investigating the way in which Swiss mental health care professionals perceive and resolve the dual loyalty conflict.

## Methods

This article follows the “Journal article reporting guidelines” for qualitative research by [23]. Further, we follow the recommendation of Tran, Baggio [24] and describe the population at stake with terminologies such as “person with mental health condition living in detention” or “incarcerated person with mental health condition”, they are used interchangeably.

### Study design

This qualitative study is part of a larger Swiss-wide research project on mental health of older persons in detention (*‘Agequake in Prisons –second part)*. As part of that project, we not only gathered qualitative data from mental health professionals (described below) but also from older incarcerated persons, as well as quantitative information on their mental health condition from medical records and standardized surveys. As older persons in prison are a minority and there is little data on the mental health of this population [25], the overall goal of the qualitative data collection was to gain insights into their experiences on aging in prison, living with a mental disorders, and their perspectives on prison mental health care. As these are complex social processes that we, to date, know little about, we applied an explorative qualitative approach to capture these social phenomena. TW and BE conceptualized the research project. Both have many years of research experience on the topic of older incarcerated persons as well as in employing qualitative methodology [15, 16, 26–28]. Two research assistants completing their doctoral education conducted the interviews. They were trained in qualitative data collection and received supervision throughout the data collection process. Ethics approval was obtained from the regional ethics committee (Ethikkommission Nordwest- und Zentralschweiz) and from the local ethics committees.

### Data collection

Face-to-face interviews were conducted between April 2017 and January 2018 with mental health care professionals working with incarcerated persons. We applied convenience and purposive sampling in order to include opinions from professionals with diverse backgrounds. We included mental health professionals with a background in mental health (psychiatry, psychology, and psychiatric nursing) working with incarcerated patients and a minimum of 10 years work experience. We contacted MHPs working at psychiatric clinics that house forensic units and forensic psychiatric services that provide mental health care to correctional institutions (for more details on the study recruitment procedures, see Table 1). Data analysis was conducted along the on-going data collection. Thus, we were able to identify when data saturation was reached and were able to include more participants if needed. We identified data saturation applying the principles presented by [29]; the ability to obtain additional new information has been attained, further coding is no longer feasible, there is enough information to replicate the study.

**Table 1:** Participant characteristics

	German speaking language region	French speaking language region	Italian speaking language region	Total
*Recruitment scheme*				
Mental health professionals *invited* for partipation	N = 35	N = 31	N = 2	N = 68
Mental health professionals *declined*	N = 19	N = 18	N = 0	N = 37
Mental health professtionals *participated*	N = 16	N = 13	N = 2	N = 31
				Response Rate: 45.6%
*Participant characteristics (N* = *29; 2 excluded from analysis)*				
Sex				
Female	N = 3	N = 4		N = 7
Male	N = 13	N = 7		N = 22
Professional background				
Psychology	N = 5	N = 1	Excluded from analysis	N = 6
Psychiatry	N = 11	N = 6		N = 6
Psychiatric nursing	N = 0	N = 6		N = 6
Institutional context				
Forensic-psychiatric institutions	4	1		N = 5
Psychiatric-psychological saervices	6	3		N = 9

We completed 31 interviews in the three major language regions (German, French, and Italian speaking). At the time of data analysis, we decided to exclude the two participants from the Italian speaking part of the country since they were not involved in providing mental health care. This resulted in 29 interviews with mental health professionals with experience in the treatment of persons with mental health conditions living in detention. Please see Table 1 for details on the study recruitment process and participant characteristics.

All participants were first contacted via email or phone, then they received information about the study and the informed consent form by email before the interview, and finally they were interviewed personally by the researchers. At the scheduled time and place of the personal interview, the researchers explained again the purpose of the study, specified that all data was treated confidentially and reminded that refusal to participate was possible at any time. Thereafter, written informed consent was obtained. There was no compensation provided for study participation.

**Fig. 1:**
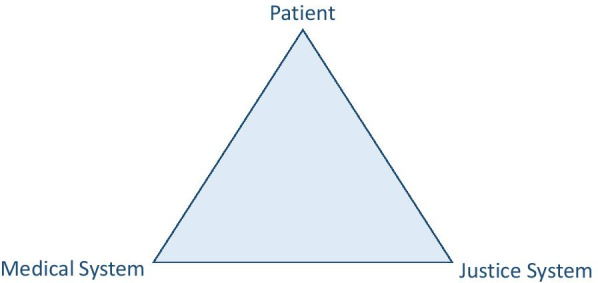
Elicitation technique

An interview guide developed for the purpose of this study guided the discussions with the study participants (see Additional file 1 for the full semi-structured interview guide). The open-ended questions within this interview guide covered topics on mental health care in prisons while specifically probing on role conflicts and dual loyalty issues that are inherent to the position of a mental health care professional providing court-mandated treatments. Opinions on the dual loyalty conflict were additionally encouraged through the use of an elicitation technique, which consisted in asking the mental health professionals to position themselves using a coin within a triangle that we constructed to represent this conflict (see Fig. 1. Elicitation technique on dual loyalty conflict). The use of this triangle graphic reflects the idea of the dual loyalty that they may perceive: (a) between them being agents of the healthcare system towards patients who are mandated to seek therapy, and (b) between their patients and the society (judicial side) (see 1). Therefore, the dual-role conflict is a result of this triangular relationship as the clinician has to take up two roles: treating the patient to ensure his rehabilitation into the community and evaluating the patient’s risk to the society, thereby enabling the justice system to continue incarcerating the patient. Both roles come with different sets of norms, which at times, can be contradictory from the perspective of a healthcare personnel. Further, we did not define our understanding of the triangle to the participants but targeted at eliciting their perception of this triangle. Hereby, we aimed at shedding light into their personal understanding of dual loyalty conflicts and their actions taken from this. They were specifically asked to reflect on inpatient involuntary treatment orders and to separate this from other kinds of mental health treatments with incarcerated persons.

The interviews took place in person and were conducted by two research assistants (either HM or SH). They were trained in qualitative interview techniques and were working on their doctoral degree at the time of interviews. Interviewer and participant met the first time on the day of the interview, thus, there was no relationship prior to data collection. Only one interview meeting took place with each participant and no repeat interview was done. Interviews were held in the language spoken by the participant, either French, German or Swiss German. Thereafter the interviews were transcribed verbatim in the language of the interview, except for Swiss German interviews, which were transcribed in Standard German. Swiss German is a spoken dialect and it is common practice to use Standard German in writing. The interview length ranged from 48 to 90 min, with an average of 67 min. All interviews were audio-recorded upon the consent of the participant and transcribed verbatim, paying particular attention to the anonymization of the information collected.

### Context of court-mandated treatment settings

In light of our analysis, it is important to outline the Swiss correctional context to illustrate potential areas in which mental health professionals might be affected by dual loyalty conflicts.

The Swiss Criminal Code (SCC) distinguishes between custodial sentences and so-called “measures”. If the crime stands in connection with a severe mental disorder, a person can be sentenced to a “therapeutic measure” on the basis of a thorough forensic psychiatric assessment (Art. 59 ff. Swiss Criminal Code (SCC)). According to the law, a therapeutic measure can be pronounced only if it can be expected that this sentence will divert the person from committing new offenses related to the mental disorder diagnosed in the assessment. Although responsibility is usually diminished or abolished when the above-mentioned criteria is met, this is not a *sine qua non* condition for the judge to order a therapeutic measure.

Adults can be sentenced to inpatient psychotherapeutic treatment to treat mental health issues (Art. 59 SCC) or substance use disorders (Art. 60 SCC) or outpatient treatment (Art. 63 SCC). A person is sentenced to a security measure (Article 64 SCC) when the mental health illness connected with the crime is considered especially severe and potentially ‘untreatable’. In these cases, issues related to treatment remain in the background, the main concern being public safety.

Key differences in relation to mental health care between Swiss custodial sentences and measures are related to placement, treatment, and release conditions. First, persons convicted to a custodial sentence are housed in correctional institutions, which include open and closed regimes. Persons sentenced to inpatient therapeutic measures should be placed in so-called “therapeutic measures centers” or forensic-psychiatric units or institutions. An outpatient therapeutic measure can take place either in the community or in a correctional institution. In reality, they are often placed in ordinary correctional institutions due to the lack of specialized facilities, especially in the French and Italian speaking areas of the country. Second, treatment conditions for persons sentenced to a custodial sentence should follow similar standards as in the community (e.g. in relation to medical confidentiality). Persons sentenced to a measure receive mandatory treatment, which is thus involuntary. Further, medical confidentiality is limited as the authorities expect regular reports on the person’s mental condition and therapy progress. Third, persons sentenced to a custodial sentence have a definite end-date to their imprisonment while a person sentenced to a “measure” has no definite end date. The reason is that even though these measures are time-limited, they can be prolonged repeatedly (with the exception of Art. 60 SCC, which can only be prolonged once). Their release depends on their mental health and their progress in therapy, which includes their risk of reoffending.

Psychotherapy sessions with persons mandated to stationary treatment take place within the institution. The fact that a mental health professional works in prison can be seen as a reason enough for a patient to doubt his or her professional independence. Further, mental health care professionals are through their physical ties in touch with other professionals. Depending on the setting, the extent and types of interactions with other professionals vary. For instance, mental health care professionals working in a forensic-psychiatric institution will be part of the team and in regular exchange. Others who work in a correctional institution might only “come in” for psychotherapy session and might not be part of the team. Their exchange with other prison staff will therefore be to a lesser extent. The treating mental health professionals have to provide at least yearly a report on their patient’s mental health and therapy progress to the authorities. Their relation with the authorities might range from sending a written report to the authorities to personal encounters with the person responsible. For instance, in some parts of the German speaking language region, the authorities participate at yearly treatment planning conferences, during which also the patient is also being heard. Thus, dual loyalty conflicts can arise from the interactions with prison staff, the authorities, and other staff subordinated to the judicial system. The nature of these dual loyalty conflicts might differ depending on the type of institution and the specific treatment conditions in each setting.

Current practice of training and specialization of mental health professionals working with court-mandated patients is diverse. Most mental health professionals working in correctional and forensic-psychiatric institutions have a background in general psychiatry and psychotherapy. Further specialized education is often provided on an institutional level. A specialist title can be acquired for forensic psychiatry, psychology, and forensic nursing.

### Data analysis

Data were processed using the software program MAXQDA. Our analysis was framed within the thematic analysis approach [30]. In order to build a uniform coding tree, eight interviews were first read and coded together by five project members. This allowed the study team to discuss different nuances that were visible in the data and to reach consensus on the dimensions identified by each code, its name and its definition. Thereafter, three study team members (HM, SH, TW) individually coded all the remaining transcripts and came together to discuss the new codes, solve disagreements, and sorted the final coding tree. All analysis took place in the language of the interviews.

Taking into account the richness of the information collected and the broader scope of the interviews, only coded data related to dual loyalty and the elicitation technique were extracted and examined in this paper. HM carefully read this data segment in its entirety and reanalyzed them according to the purpose of this study. This in-depth examination of one topic was also conducted applying thematic analysis. The results were discussed with all the co-authors. They are presented following to major themes “Where do I align myself? Understanding dual loyalty” and “Solving dual loyalty conflicts and the therapeutic alliance concerns”. Both topics are further divided into subthemes, which are the outcome of the researchers’ agreement on the key issues relevant to the issue of dual loyalty. In the results below, we use PD to refer to our participants from the German-speaking part and PF represents our participants from the French-speaking part of the country. The quotes presented in the results sections were translated into English after completion of the data analysis. HM translated the codes from the original language into English, the translations were checked by an English native speaker.

## Results

### Where do I align myself? Understanding dual loyalty

#### Justice system, health system or both

The elicitation technique was successful in provoking responses linked to dual loyalty conflicts that mental health professionals face while working in court-mandated treatment settings. They found the use of this technique illustrative of their situation: “It’s exactly this triangle that you plotted. Yes, we are part of a triangle…” or “Well, these are of course those fundamental conflicts we deal with in this job.”. Participants stated that within this triangle of mutual dependence among the patient, health care providers, and the justice system, nothing works without the other parties involved. The participants described the nature of this interrelatedness somewhat differently.

For instance, a few participants would take up a position in the middle of the triangle because they perceive themselves as accountable towards all players and having to integrate the differing demands. They also characterized the interrelatedness as a work collaboration in which each player has to fulfill his or her role. Another participant also positioned himself in the middle due to his management position, which places him/her as a go-between from the mental health professionals to the justice system: “There in the middle. Committed to all three.”. Another stated: “We try to integrate it all as a whole, so that’s why I positioned myself in the middle and because we are really working in collaboration.”. Elaborating on the position in the middle, one participant explained: “Well, on the one hand you are a representative of the health system, on the other hand you try to be there for the patients and then also again you are working with the justice system and are also part of this system. I think with the middle it is just right.”.

The other respondents tended to privilege one of the sides of the triangle and positioned themselves a bit closer to the medical system, the judicial system or the patient. However, the differences in these positions cannot be linked to a particular stance taken. Several interviewees reasoned that they understood themselves as being representatives of the medical system in light of the caregiving role that they had, but inevitably connected with the justice system due to security imperatives and the crime committed by their patient:

So I'm part of the medical system, so I'm, uh, I'm integrated into the medical system, I'm paid by the medical system, uh, that's clear, but it's true that when you work in the field of forensics, and that's what it is [...] the specificity of forensics—the judicial field—is not accessory.

I'm a player of the health care system, so first of all, I'm going to refer to the system to which I belong. ..., we have to address security imperatives, which are what they are, we can't just do anything, there's the crime which has its place in care taking, which are things that we address.

Others underlined that the justice system is the sponsor that pays for treatment, defines its goals, and provides the framework for the therapeutic setting. For instance, one participant said: “I am commissioned by the judiciary to look after the health of these patients; yes, within the framework of the judicial system.” This accountability to the justice system was described as a responsibility to the general population, towards safety of society: “Of course [there is] also an accountability towards third parties, precisely the justice system or somehow also towards the general population.” and “It's about protecting the community but, at the same time, protecting the community, the patient is also part of the community.” It is relevant for our analysis to point out that the respondents’ positions in the triangle were not systematically different between the participants of the two language regions. The only trend was that no French speaking participants positioned him-/herself on the side of the justice system. The majority of participants, however, considered that their job is to care for the mental health of their patients while trying to integrate the demands from all the players involved. This vision was irrespective of the stance they took and the language region they came from. Nevertheless, they considered that the accountability towards the justice system affected their therapeutic practice with the patient because of the security needs that arise when one works in corrections.

Only two participants took extreme positions, and they represent exceptions to the prevailing stance presented above in relation to the conflict between the well-being of the patient and that of the society. One interviewee from the German speaking region positioned himself close to the patient but on the side of the judicial system. This expert described the well-being of the individual as a nice side-effect of the risk-reduction goal. He/she noted that the goal of risk reduction is above the goal of individual well-being: “The main goal is the victim-prevention. If in doing so, the person becomes healthier and happier, then that’s all right with us but it is not the declared goal.” Another respondent from the French speaking region claimed that the only mandate was to take care of the patient’s health. According to this position, mental health professionals should never give their opinion on safety and security aspects: “The doctor often gives an opinion on aspects that are not medical, [but] he is there to care for the person, he is not there to give an opinion on the dangerousness.”

#### Situational factors influencing one’s own positioning

Many respondents manifested that it is very important to be aware of the different players, as well as of the influences and demands, in order to deal with the dual loyalty conflict. They considered that it is crucial to have an explicit and clearly communicated standpoint towards the patient, and to be realistic about one’s own role, position, and expectations. Underscoring the challenges of this clear standpoint, one participant noted: “This sometimes causes great difficulties and requires us to be extremely transparent towards everyone. And it requires that we are also realistic towards ourselves/ that we are realistic as well.”

The study participants further reported that professionals working in a correctional context would position themselves towards the justice or the medical system differently than their colleagues. This difference was described as a consequence of the basic conflict that mental health professionals tend to resolve in differing ways.

Institutions for court-mandated treatments always fluctuate/oscillate between the [two] poles, am I more on the treatment pole or more on the security pole? This is the kind of tension that we always have to manage and that also tilts, and some employees are more on one side, some more on the other and the professionally demanding [issue] is to practice both: To guarantee sufficient security and to give importance to individual needs of each patient within the scope of the possible.

Nonetheless, the triangle was depicted as a dynamic model in which one’s own position changes according to situational factors. “Sometimes you are more here, sometimes more there, sometimes more there… But you always have to go back to the middle to look: Where am I positioned?”; “Time and again, there are moments in which you're just kind of swinging, and in limbo.”

Some respondents hesitated when asked to position themselves due to the many possible situations that they would face in their work life. “It’s hard to say because I see the whole, right? It needs everything (8 s. pause). Yes, you have to… there are so many situations!”. A few participants pointed out that while directly working with the patient, they would position themselves very close to him/her. The justice system would sometimes even slip into the background: “sometimes the side of the authorities gets forgotten because of therapeutic alliance. The skill is to obtain a good therapeutic relationship although you always have to take the authorities into account.”; “For example when I therapeutically work with the patient, then I move here to the patient.” However, whilst writing the report to the justice system, some participants would feel themselves further away, taking a step back from the patient-centered position to be able to assess the patient from an outside perspective. “If, for example, I have to write a report, then I am part of and perceive myself as part of the justice system. There I have to look at the patient from […] outside [the patient-physician relationship].” The same respondent highlighted end-of-life situations as an example of a case in which the justice system becomes less important, as the quality of life and a dignified death of the patient are central.

#### The burden of dual loyalty

The ethical, but also administrative, burden that dual loyalty poses on the mental health professionals becomes apparent in the following two examples. One respondent expresses his envy towards the chaplains in this way:

Sometimes I am a little jealous of them. They can… they do very good work but they don’t have the obligation to document it, they never have to write a therapy report and they are not involved in the triangular relationship – authorities, therapist, patient – that you have with the patients in court-mandated treatments. When we do therapy with patients in court-mandated treatments we are accountable to the authorities. And we have to regularly write therapy reports, the chaplains don’t have to do all that.

Another respondent pointed out that mental health professionals often feel as being the only ones that the imprisoned patient can trust, even if the latter is aware of their accountability to the justice system. One interviewee expressed his/her wish of incorporating professionals from institutions with no attachment to the justice system as counselors for the incarcerated persons.

That the burden is not born as one-sidedly as we sometimes feel, it would be helpful if there was another player who would then also actively defend the rights [of incarcerated patients] in the sense of an assistance relationship [to the incarcerated patient].

### Solving dual loyalty conflicts and the therapeutic alliance concerns

#### Motivating the patient: seeing the benefits of the treatment and its goals

Study respondents differed in the way they prioritized their two missions of ensuring patient health and protecting public security. They highlighted it as a specific challenge of their work in light of the need to integrate and balance the contradicting demands arising from both missions. Some respondents resolve this conflict by predefining the goal of risk reduction for the patient, stating that it is in the patient’s own interest that he/she does not reoffend. Thus, they combine the two missions in a single treatment objective: “It is actually in his interest that he does not produce victims and that he does not do dysfunctional things that harm other people.” Noting the same point, another participant put it as:

If one is really interested in the patient, in the humanity [issues] which are part of those patient cases, we have to guide them to regain their humanity. That means not commit an offence anymore, diminish the risk of a relapse. And one cannot do this work ignoring the justice system.

These participants accept that their patients will not always agree with that agenda, but they nevertheless try to motivate them insisting on the fact that the treatment is in their own interest and will help them not only to enhance their mental well-being but also to ensure that they are less likely to reoffend. They further pointed out that the mandatory aspect of therapy can often act as an initial means to motivate the patients to participate in the treatment. They consider that, if they manage to build a therapeutic relationship at the beginning, then the mandatory aspects will later occupy a second role, as patients will want to engage in therapy out of their own volition.

That strongly depends on the motivation. If a patient says: ‘Yes, I know I have a problem, I want to change something’ then one can… Once a year one has to write a report, then that works. If someone comes because he is obliged to and [he has] no personal initiative at all, no understanding or something like that, then of course it is extremely difficult to find an alliance, an access in the first place.

#### Building trust and therapeutic alliance

Irrespective of the positioning within the triangle and the alignment with either the justice or the medical system, all participants agreed that the objectives can only be achieved through the construction of a strong and trustful relationship with the patient. Participants claimed that they have to provide therapy, which is not possible without the patient’s collaboration. Respondents also highlighted mental health professionals have to be committed to the patient and show a certain dedication to build trust and a therapeutic alliance. This is succinctly expressed by one participant: “But it is the patient, it is him who is in the centre of everything.” Thus, even though the priority of treatment goals may differ, they concurred with a need for a strong therapeutic alliance and underlined that it is not possible to reach any goal without being devoted to the patient. One participant described this point in the following way: “One must not forget the patient. If the therapeutic alliance is not there and the trust is not there – and of course it also needs a certain commitment to the therapy – then one cannot do therapy.”.

Furthermore, participants pointed out that a cornerstone in developing an alliance is to win the patients’ trust. They stated that patients are generally apprehensive of opening up to them due to their concern about the mental health professionals acting in the interest of the justice system. Their clients would be particularly worried about the mental health professional’s assessments and about the limits of medical confidentiality. Participants underlining these fears stated: “…trust in the therapist, that he/she is not just a ‘police agent’ who may be trying to unmask other crimes. Well, those are the concerns.” “Fear of being dependent on the judgement of forensic experts, which is generally always bad with prisoners—this is the prejudice, which is pronounced in court-mandated treatment settings.”

#### Transparency as a technique to establish therapeutic alliance

Several respondents spontaneously pointed out that one of the fundamental questions is whether it is at all possible to establish a therapeutic alliance in the context of a court-mandated treatment.

And then, of course, there is always the question of how far it is possible to build up a so-called trusting therapeutic relationship within the framework of a court-mandated treatment, if the patient always knows that the legal system sits breathing down one’s neck, and anyway, it is unfavorable for me if I tell during therapy that I had a relapse.

The vast majority of participants emphasised the importance of transparency in order to resolve this conflict. They highlighted that it was crucial to be very clear, realistic, and open about the conditions. This strategy was perceived as being well-appreciated by the patients and also as a main driver in motivating the patient. “We have not only, but mostly made good experiences if we say that honestly at the beginning.” “I think this is an essential point to motivate the patients to engage in their therapy. That they understand as well as possible what is actually going on.” Transparency was further specified to be different from shared decision making, i.e. the patient will not have the right to decide which information is transmitted to the authorities and which clinical aspects will remain confidential: “So, here knowledge plays a role and transparency, but not a codetermination in what happens with the information.” The most common example of being transparent mentioned by the interviewees from both language regions is that mental health professionals should provide the patient with some insight about the report they have to write for the justice system *before* that report is sent off. “We tell him that we will write a report about him, we show it to him when it is written, but he does not choose what goes in.” “Here, we have a very open attitude. We also hand over the reports that we write to the prisoners.”

Several respondents from the German speaking region stated that on-going feedback and direct feedback towards the patient is important during the entire therapy process, and not only when the report is being written. One respondent even explained that they would include the patient in the meetings with representatives from the justice system: “And the patient is then also heard in these meetings, about his wishes and ideas”. Another reported:

We also constantly say how we perceive it. So, if you think someone sticks to the surface, for example, and doesn't go into depth, then we say it. Not just after a year, when we have to write a report, but constantly.

Study participants from the French speaking region pointed out that they would only provide a detailed report to the justice system if the patient releases them from their medical confidentiality obligation. However, they would inform the patient about the negative consequences that they might face if they refuse to make the report available.

If the patient doesn't authorize us to give the information to the authorities, we simply tell the authorities that we are working with this patient but that he doesn't authorize us to inform them. So that's it. It's negative for the patient, that's how it is. But it's very rare that the patient refuses. Normally they are ok with the fact of informing the authorities. We explain to them that it's really to their advantage.

Another respondent from the French speaking region gave an example of lack of transparency and the problematic consequences if the patient is not aware from the beginning of the therapy that the mental health professional has the legal and ethical obligation to inform other prison staff of risks such as patient’s propensity to be aggressive.

That we have not had the time to warn him, it’s just a question of sequence – if there was a danger, I… I think that we always have to warn the patient ‘listen, there I do not agree; you cannot tell me this, things like that’ finally there is a violence that has surged, it is necessary to work on where this comes from, what is happening, but we cannot let this information remain ‘passive’ – [that is] we cannot be the receptors and be the silent witnesses of a hetero-aggressive risk.

Overall, participants agree that the specific challenge of their field is to build a trustful therapeutic alliance in spite of the dual loyalty conflict, and that transparency is the key factor to overcome that challenge. “But it is the special challenge in our job that we nevertheless have to find a good access under these conditions. And this works mainly through transparency, the patients must know exactly what is communicated, when and why”.

#### Transparency when communicating with representatives of the justice system

In line with this dual loyalty conflict, many interviewees highlighted the importance, but also the challenges, of their exchanges with representatives of the justice system: “The exchange, these interface functions between medicine and justice, are a very central part”. Similarly, another participant noted: “We work together with/around the same people […]. So, it is always important to be able to communicate about the situations. It is a skill to communicate about situations without disclosing medical secrecy”.

The interviewed mental health professionals stated that the dual loyalty conflict makes them face difficult decisions when choosing the kind and the amount of information that they share with colleagues from non-medical professions. They reported that a dilemma was created by their obligation to share important information with other professionals while assuring trust and confidentiality towards the patient. This was highlighted by two participants:

This is the dilemma which we are facing when we treat mandated patients that we are not allowed to withhold important information.

Our duty as health professionals is to preserve our working instrument, which is trust, which the patients have in us, if we tell everything to the authorities, we will lose their trust.

Several respondents insisted on the fact that it was hard to decide which content to pass on and in what detail. This poses a challenge when writing the report for the justice system but also in everyday work life: “Basically, we always have to weigh up which things to report, what goes into the report”. Describing the personal decision-making process in securing to ensure confidentiality, a participant stated: “You have to spend time justifying yourself … you have to be well positioned in your role, it's not always easy because you have duties concerning medical confidentiality. What should we say, what shouldn’t we say, what should or shouldn’t we share”. Furthermore, another participant, noting the dilemma, added that he/she discusses it with the patient:

What is of course a recurring topic is this delicate balancing act with regard to confidentiality. So, how much exchange is there, how much may I say, may I not say, which of course also has to be discussed with the patient.

Several respondents considered that there should be an on-going exchange with the prison staff and that transparent communication with all players was crucial: “This sometimes causes great difficulties and requires us to be extremely transparent. Towards everyone”. Others agreed that the information should not contain intimate details in order to protect the privacy of the patient, but it should cover the gist of it.

There is some exchange, but there are limits. I personally think it needs the possibility that you exchange without going deep into the content. That I do not say anything about the therapy, but that I say: ‘At the moment he is not doing so well, you should have an eye on him’.

## Discussion

This qualitative interview study is unique as it addresses the insufficiently researched question of how MHPs working in court-mandated treatment settings perceive and resolve their dual loyalty conflict**.** In doing so, we were able to gather new qualitative data about practical ways used to solve this ethical dilemma. Not surprisingly, our study results indicate that mental health professionals differ in the way they perceive their role, obligations, and responsibilities. These different perceptions were expected in light of the diversity in terms of cultural, social and personal background that characterizes our sample. As pointed out by Niveau and Welle [1], forensic psychiatry is a field of dual ethics, which forces mental health professionals to choose a stance.

From a legal point of view, the problem resides in the fact that mental health professionals are simultaneously caring and controlling. The MHP has the obligation to reveal all *significant* information to the authorities, and this overrides medical confidentiality that characterizes a classical therapeutic relationship. The crucial question is to define which bits of information are *significant*. Some clinicians consider that all the information must be communicated to the authorities, while others think that communicating the conclusions is sufficient and that details are not required. Our study shows that not only this theoretical difference about the level of detail that must be communicated to make a report credible for judicial authorities, but also situational factors influence the positioning of MHPs and drew them at times either closer to the security or the treatment pole. These theoretical and situational factors affect therapeutic practice in relation to medical confidentiality and also in terms of the definition of treatment goals.

Against our expectations, the positioning of participants from the two language regions did not systematically deviate. Moreover, even when participants’ personal standpoints were theoretically different (i.e. some practitioners from the German speaking region stated that they worked for the criminal justice system), in their daily clinical practice the majority of participants showed that they work in the interest of the patient’s well-being, while trying to integrate security imperatives. The ethical dilemmas resulting from this conflict convene into questions on how to develop a trustful therapeutic alliance while sharing critical information with representatives of the justice system. The one element that all participants consider essential in promoting a high-quality alliance with the patient was transparent communication.

The importance of transparency is in line with major recommendations on how to solve dual role dilemmas such as the guidelines provided by the American Academy of Psychiatry and the Law [5]. However, the responses of our participants show, that being transparent about the limits to confidentiality and the conditions of court-mandated treatment, do not resolve the dual role conflict, per se. However, it might facilitate the development of a therapeutic working alliance in spite of the dual loyalty conflicts. Transparency to establish and maintain the alliance could therefore be as a tool to act as a double agent. As highlighted by Stone [31], when a relationship is established, the client might forget that he/she has been warned about possible dual loyalty. Thus, the perceived burden of this dual-role conflict and ethical dilemmas resulting from it must be solved by the clinician. In light of the lack of practical guidelines, questions remain on, for instance, the degree of completeness of the information that the authorities must receive and other prison staff or how to convince the patient that the predefined goal is in his/her best interest. This suggests, at least indirectly, that there is a need for more detailed practical guidelines as well as training for clinicians working in this field. Nevertheless, as transparency in relation to the therapeutic alliance was put in the spotlight by our participants, we consider it more closely in the following paragraphs by discussing transparency in relation to (a) building therapeutic alliance; (b) truthful exchanges about treatment goals; (c) building trust with patient; and (d) communication with the justice system.

Transparency was considered valuable not only from an ethical point of view regarding the patients’ right to receive all the information concerning him/her; but also from a consequentialist point of view concerning the best therapeutic outcome. Indeed, the therapeutic alliance is significantly linked to positive outcome measures of psychotherapy [32]. Little is known about the therapeutic alliance in mandatory treatments, as the majority of research is based on general psychotherapy. Nevertheless, it is assumed that results can be mirrored to the coercive setting as it has been shown that alliance ratings are independent of the patient’s legal status [33]. A further declaration in this respect is the therapeutic alliance being one of the common factors in psychotherapy that are independent of the technique used [34]. The widely used concept of the therapeutic alliance was established by Bordin [35]. This trans-theoretical construct comprises three dimensions: Goals, Tasks, and Bonds. Meyer, Hachtel [36] state that the three dimensions are affected in court-mandated treatment settings due to aspects such as the therapists’ dual role. However, little is known on the therapists’ strategies to resolve these influences in daily practice [37].

Transparency, besides its ethical value, is also the prerequisite to truthful exchanges about treatment goals; therefore, it is also associated with improved therapeutic relationships and positive treatment outcomes. Mutual agreement on treatment goals is highlighted as one of the key factors in developing and maintaining a therapeutic relationship between patient and therapist [35]. Mandatory treatments add a unique challenge: The majority of the study participants agreed that the involvement of the justice systems brings along a predefined treatment goal: the prevention of recidivism. Hence, the patient is forced into a psychotherapeutic and psychiatric treatment with a goal imposed by a third party. Respondents used two approaches to resolve the conflict of facing a pre-defined treatment goal.

First, realizing that it is not possible to develop treatment objectives jointly between the patient and the therapist without external influence, the strategy privileged by the practitioners of our sample is to try to convince the patient of the benefits of the predefined goal, that is, to see the advantages of not committing another offence. This means that they try to motivate patients to accept the treatment objective in order to resolve their dual loyalty conflict. Some authors have argued that the agreement on therapeutic tasks and objectives is of minor importance in mandatory settings because the main goal of the inmate is to recover his/her freedom [36].

Second, the majority of all respondents stressed their role in caretaking and focused on their patient’s well-being. This attitude is in accordance with the recent paradigm shift towards greater integration of strengths-based approaches to increase the effectiveness of treatment [38, 39]. In line with this, MHPs’ psychotherapeutic techniques emphasize, next to risk management, also the patient’s individual needs, protective factors, and personal strengths—thus respecting their autonomy [40]. With regard to treatment goals and the therapeutic relationship, although the overall objective is pre-defined, the path to reach this goal is negotiated. Thus, tasks and sub-goals are mutually developed and agreed upon.

Furthermore, transparency is a crucial factor for building trust with the patient. Trustworthiness has been depicted as a therapist characteristic that promotes strong rapport building with clients and that can be complemented through the application of a series of techniques that convey trust [32, 41]. Patients’ trust in their treating therapist is positively linked to health outcome measures and has therefore been described as one of the foundations of effective treatment in health care [42].

Medical confidentiality ensures trust and protects the patients’ private sphere [15]. However, confidentiality in court-mandated settings is limited, and that threatens the patient’s trust. Mistrust was described as a key issue when working with mandated patients by numerous study participants. They concurred that the main and most important tool to build trust and to develop a therapeutic alliance was transparency. Most participants stated to be very clear, realistic, and open about the conditions of mandated treatments as well as of the limits to medical confidentiality. They emphasized the importance of constant feedback and authentic communication with the patients. Professionals in the field of forensic psychiatry have underlined the importance of transparency regarding the conditions of limited confidentiality [17, 38, 43] which has also been recommended by “Ethical Guidelines for the Practice of Forensic Psychiatry» provided by the American Academy of Psychiatry and the Law [5]. Empirical research substantiates that it is crucial to be open about the MHP’s dual role in treatment and control [8, 37]. Our findings are therefore in line with previous research and provide further evidence that transparency is a key factor to develop a therapeutic alliance in coercive treatment settings.

Transparency when communicating with representatives of the justice system was also underlined as key element by the study participants. However, in this case the main challenge is to decide whether to share all the information available or only the gist of it, leaving aside the details. Participants argued that it is crucial to protect the privacy of the patient but also to consider security aspects and to prevent risks for prison staff. It was perceived as particularly challenging to decide what piece of information to share while trying to integrate and balance the contradicting demands arising from both objectives, patient health and public safety. This leads to an ethical dilemma that clinicians have to resolve in their daily practice, when they are confronted to situations in which the have to prioritize one over the other. The lack of guidelines on how mental health professionals should breach confidentiality in specific situations has been highlighted previously [44] and our interviews corroborate its importance. It is not surprising to see that the members of our sample perceive this as burdensome and resolve these conflicts differently in the absence of such practical guidelines.

Taken together, our findings suggest that dual role conflicts in court-mandated treatment settings are still a pressing issue for mental health professionals. Based on our findings, it is too early to provide specific recommendations for clinical practice. However, we believe that this bottom-up approach has the potential to identify typical situations that result from the dual-role dilemma, based on which practical guidelines could be developed. On an institutional level, we therefore recommend to follow, for instance, the “moral acquaintance procedure” proposed by Ward and Ward [45]. This approach aims at delivering concrete procedures for dealing with dual role conflicts in practice. This way, we could advance our knowledge and awareness on dual loyalty conflicts, stimulate the discussion on possible strategies to resolve ethical dilemmas, and support the individual practitioner in their decision-making.

## Limitations

We applied a qualitative study design that is of an explorative nature and we recruited our sample through convenience sampling. Moreover, the sample comes from a single country (Switzerland) and consists of mental health professionals that work with mandated patients in closed settings, consisting mainly in prisons, forensic, and therapeutic units. These facts threaten the internal and external validity of our results.

In terms of internal validity, a convenience sample means that the stakeholders that were interested in participating might have had a specific set of opinions that influenced the study results. One can never exclude the influence of the institutional regulations and cultural mindsets that prevail in their environment. Similarly, they might have had advanced opinions about what is correct and socially acceptable for a person in their position. Following the classis rules for research of this kind, we tried to limit the influence of social desirability by assuring anonymity and confidentiality.

Further, the mental health professionals interviewed work in different treatment environments including prisons, therapeutic measure centers, and forensic-psychiatric units. The work and treatment conditions therefore vary with the setting, which potentially influence the experiences of dual loyalty conflicts. This might have caused some heterogeneity in the participants’ responses. However, the treatment settings within Switzerland differ widely, even for persons living with similar mental health conditions sentenced under the same article. One reasons for this is criminal law being national law but the execution of sentences being under the responsibility of the individual states. The organization of the institutions is consequently very different across the country. For instance, the French speaking language region lacks places in forensic-psychiatric hospitals. Mentally ill persons sentenced to a therapeutic measure are therefore more frequently placed in correctional institutions compared to the German speaking region. Furthermore, the healthcare in prisons of the French speaking region is under the responsibility of the health department while some health services in the German speaking region are under the responsibility of the justice department. Thus, to capture the variety of experiences on dual loyalty conflicts in Swiss psychotherapeutic treatment settings, we decided to include mental health professionals who work in all different types of settings.

In terms of external validity, the results cannot be directly generalized to other countries and other settings. It must be mentioned, however that the study covers two different linguistic and cultural regions, which pleads for some possibility of generalization. More difficult is to establish whether the findings in the specific setting studied can be transposed to countries where the organization of mental health care and the basic conditions of treatment are diverse, which is often the case even within the same country. However, the ethical dilemmas and the daily clinical decision-making for practitioners working in closed-settings are rather universal, which pleads for at least a limited use of our results for studies conducted in that kind of setting or with involuntary treatment orders in general.

Finally, the mental health professionals’ triangular relationship has implications on multiple dimensions. Our study participants elaborated mainly on topics such as therapy goals and limited confidentiality in involuntary treatment orders. However, other situations such as mental health professionals acting as expert witness at court, or being directly involved in the application of punitive measures, have not been addressed by our participants because they were not the subject of this study. Other topics such as the involvement in custodial activities or the use of sedatives for security reasons has not been the focus of our participants’ responses. Further research should investigate the clinicians’ decision-making processes in other circumstances in which they are affected by dual loyalty conflicts.

## Conclusions

Mental health professionals working in court-mandated treatment settings are obliged to resolve ethical dilemmas in their daily practice. Transparency seems to be the crucial factor when communicating with the patient and with representatives of the justice system. More specifically, patients need to be informed from the beginning of therapy about limits to confidentiality. It is also recommended to develop guidelines that define the level of detailed information that should be disclosed when communicating with the authorities of the justice system. The study findings show that there are certain techniques and approaches that are applied by the majority of mental health professionals working with mandated clients, and that these techniques and approaches are independent of the side taken in the ethical conflict known as dual loyalty within forensic psychiatry. We therefore call for more research on common factors of psychotherapy in court-mandated settings to advance guidelines that support clinicians in their daily decision-making. This would alleviate the burden that is posed on mental health professionals by the dual loyalty conflict and is therefore of utmost importance for clinical practice.

## Supplementary Information

**Additional file 1**. Semi-structured interview guide.

## Data Availability

The dataset analysed during the current study is not publicly available. Our analysis is based on qualitative interviews with mental health care professionals working in the forensic context. The individual privacy of our study participants would be compromised if we shared the whole transcripts publicly. However, we can share the parts of the transcripts relevant for this paper upon reasonable request.

## Abbreviation

MHP: Mental health professional

## References

[1] Niveau G, Welle I (2018). Forensic psychiatry, one subspecialty with two ethics? A systematic review. BMC Med Ethics.

[2] Pollähne H. Ethics within the prison system. In: Konrad N, Völlm B, Weisstub DN, editors. Ethical issues in prison psychiatry. 46: International Library of Ethics, Law, and the New Medicine; 2013.

[3] Goulet MH, Pariseau-Legault P, Cote C, Klein A, Crocker AG (2019). Multiple stakeholders' perspectives of involuntary treatment orders: a meta-synthesis of the qualitative evidence toward an exploratory model. Int J Forensic Ment..

[4] Sadoff RL. Ethical issues in forensic psychiatry in the United States. In: Sadoff RL, editor. Ethical issues in forensic psychiatry: Minimizing Harm; 2011. p. 3–26.

[5] American Academy of Psychiatry and the Law. Ethical guidelines for the practice of forensic psychiatry, 2005. https://www.aapl.org/ethics.htm. Accessed 8 Feb 2013. 16944556

[6] Appelbaum PS (1990). The parable of the forensic psychiatrist—ethics and the problem of doing harm. Int J Law Psychiat.

[7] Adshead G (2014). Three faces of justice: competing ethical paradigms in forensic psychiatry. Leg Criminol Psychol.

[8] Bonner R, Vandecreek LD (2006). Ethical decision making for correctional mental health providers. Crim Justice Behav.

[9] Atkinson HG (2019). Preparing physicians to contend with the problem of dual loyalty. J Hum Rights.

[10] Morandi S, Burns T (2014). Involuntary outpatient treatment for mental health problems in Switzerland: a literature review. Int J Soc Psychiatr.

[11] Graf M. Prison Psychiatry in Switzerland. In: Konrad N, Völlm B, Weisstub DN, editors. Ethical Issues in Prison Psychiatry; 2013.

[12] Pont J, Stover H, Wolff H (2012). Dual loyalty in prison health care. Am J Public Health.

[13] Physicians for Human Rights. Dual loyalty and human rights in health professional practice. Proposed guidelines and institutional mechanisms. School of Public Health and Primary HealthCare, University of Cape Town, Health Sciences Faculty; 2002. https://phr.org/our-work/resources/dual-loyalty-and-human-rights-in-health-professional-practice/.

[14] Penal Reform International. Making standards work. 2001. http://www.penalreform.org/files/man-2001making-standards-work-en.pdf.

[15] Elger A, Handtke S, Wangmo H (2015). Informing patients about limits to confidentiality: a qualitative study in prisons. Int J Law Psychiatry.

[16] Elger A, Handtke S, Wangmo H (2015). Paternalistic breaches of confidentiality in prison: mental health professionals' attitudes and justifications. J Med Ethics.

[17] Lau S, Sachs J (2015). Schweigepflicht in der forensisch-psychiatrischen Behandlung: Mythen und Realitäten. Schweizerische Ärztezeitung.

[18] Sen P, Gordon H, Adshead G, Irons A (2007). Ethical dilemmas in forensic psychiatry: two illustrative cases. J Med Ethics.

[19] Brägger BF. Massnahmenvollzug an psychisch kranken Straftätern in der Schweiz: Eine kritische Auslegeordnung. SZK; 2014. p. 36.

[20] Ward T (2014). The dual relationship problem in forensic and correctional practice: community protection or offender welfare?. Leg Criminol Psychol.

[21] Weber M (1904). Protestant ethics and the "Spirit" of capitalism. Arch Sozialwiss Sozi.

[22] Pullan B (2005). Catholics, protestants, and the poor in early modern Europe. J Interdiscipl Hist.

[23] Levitt HM, Bamberg M, Creswell JW, Frost DM, Josselson R, Suarez-Orozco C (2018). Journal article reporting standards for qualitative primary, qualitative meta-analytic, and mixed methods research in psychology: the APA Publications and Communications Board Task Force Report. Am Psychol.

[24] Tran NT, Baggio S, Dawson A, O'Moore E, Williams B, Bedell P (2018). Words matter: a call for humanizing and respectful language to describe people who experience incarceration. BMC Int Health Hum Res.

[25] Moschetti K, Stadelmann P, Wangmo T, Holly A, Bodenmann P, Wasserfallen JB (2015). Disease profiles of detainees in the Canton of Vaud in Switzerland: gender and age differences in substance abuse, mental health and chronic health conditions. BMC Public Health.

[26] Wangmo T, Hauri S, Meyer AH, Elger BS (2016). Patterns of older and younger prisoners' primary healthcare utilization in Switzerland. Int J Prison Health.

[27] Wangmo T, Meyer AH, Handtke V, Bretschneider W, Page J, Sommer J (2016). Aging prisoners in Switzerland: an analysis of their health care utilization. J Aging Health.

[28] Wangmo T, Meyer AH, Bretschneider W, Handtke V, Kressig RW, Gravier B (2015). Ageing prisoners' disease burden: is being old a better predictor than time served in prison?. Gerontology.

[29] Fusch PI, Ness LR (2015). Are we there yet? Data saturation in qualitative research. Qual Rep.

[30] Braun V, Clarke V (2006). Using thematic analysis in psychology. Qual Res Psychol.

[31] Stone A (1984). The ethics of forensic psychiatry: a view from the ivory tower. Bull Am Acad Psychiatry Law.

[32] Fluckiger C, Del Re AC, Wampold BE, Horvath AO (2018). The alliance in adult psychotherapy: a meta-analytic synthesis. Psychotherapy.

[33] Höfer FXE, Habermeyer E, Mokros A, Lau S, Gairing SK (2015). The impact of legal coercion on the therapeutic relationship in adult schizophrenia patients. PLoS ONE.

[34] Blasko B, Serran G, Abracen J. The role of the therapeutic alliance in offender therapy. In: Jeglic EL, Calkins C, editors. New frontiers in offender treatment: the translation of evidence-based practices to correctional settings: Springer; 2018.

[35] Bordin ES (1979). The generalizability of the psychoanalytic concept of the working alliance. Theory Res Pract.

[36] Meyer M, Hachtel H, Graf M (2019). Besonderheiten in der therapeutischen Beziehung bei forensisch-psychiatrischen Patienten. Forensische Psychiatrie Psychologie Kriminologie.

[37] Dowling J, Hodge S, Withers P (2018). Therapists’ perceptions of the therapeutic alliance in “Mandatory” therapy with sex offenders. J Sex Aggress.

[38] Wittouck C, Vander BT (2019). Recovery, desistance, and the role of procedural justice in working alliances with mentally ill offenders: a critical review. Addict Res Theory.

[39] Vandevelde S, Vander Laenen F, Van Damme L, Vanderplasschen W, Audenaert K, Broekaert E (2017). Dilemmas in applying strengths-based approaches in working with offenders with mental illness: a critical multidisciplinary review. Aggress Violent Beh.

[40] Ward T, Gannon TA (2006). Rehabilitation, etiology, and self-regulation: The comprehensive good lives model of treatment for sexual offenders. Aggress Violent Beh.

[41] Hilsenroth MJ, Cromer TD, Ackerman SJ. Chapter: How to make practical use of therapeutic alliance research in your clinical work. In: Psychodynamic psychotherapy research: Evidence-based practice and practice-based evidence. Totowa: Humana Press—Springer; 2012. p. 361–80.

[42] Birkhäuer J, Gaab J, Kossowsky J, Hasler S, Krummenacher P, Werner C (2017). Trust in the health care professional and health outcome: a meta-analysis. PLoS ONE.

[43] Gannon TA, Ward T (2014). Where has all the psychology gone? A critical review of evidence-based psychological practice in correctional settings. Aggress Violent Beh.

[44] Wangmo T, Handtke V, Elger BS (2014). Disclosure of past crimes: an analysis of mental health professionals' attitudes towards breaching confidentiality. J Bioethic Inq.

[45] Ward AS, Ward T. The complexities of dual relationships in forensic and correctional practice: safety vs. care. In: Zur O, editor. Multiple relationships in psychotherapy and counseling; 2016. p. 72–81.

