# Supplementary material for: Forensic mental health professionals’ perceptions of their dual loyalty conflict: findings from a qualitative study
Source: BMC Med Ethics. 2021 Sep 16;22:123. doi: 10.1186/s12910-021-00688-2 (PMC8444425; doi:10.1186/s12910-021-00688-2)
Supplement: Supplementary file 1 — Additional file 1. Semi-structured interview guide. [file 12910_2021_688_MOESM1_ESM.docx]

| **Part of the interview guide / theme / technique** | **Interview questions** |
| --- | --- |
| **Introduction** | 1. What is/was your **motivation** to work in the field of mental health care for offenders? 2. What work experience do you have in this field? 3. What involves your current position? |
|  | 1. In which setting do you mostly work with older offenders? |
| **Mental Health Care** | 1. Could you briefly summarize, the way mental health care is **organized** in your institution?    1. What are the characteristics of the treatment?    2. What is the general frequency of these treatments (per week or month), and how long do they last?    3. How is access to a MH care professionals guaranteed in case of unexpected episodes? |
|  | 1. Are the treatments provided for **older** offenders the same?    1. Does the treatment for the older differ in any way?    2. Do you notice any change in your attitude towards the elderly?    3. If you could change anything about the currently provided interventions to make it most suitable for older detainees, what would you do? 2. Could you name the three most common **needs** that you have noticed treating older offenders? 3. What do you do to address these needs? |
| **Access to Mental Health Care** | 1. Could you explain the process of how a decision is made that an offender gets in touch with mental health care staff? |
| **Role Conflict** | 1. Elicitation Technique:   Where do you position yourself? |
| **Risk Assessment** | 1. Do you conduct risk assessments of your patients?    1. Is there a certain procedure that you follow in your institution?    2. At what time points do you do them?    3. What instruments do you use? 2. Imagine you would have to explain to a student the concept of risk of relapse. How would you do it? |
